# Supplementary material for: Causal Relationship between Aging and Anorexia Nervosa: A White-Matter-Microstructure-Mediated Mendelian Randomization Analysis
Source: Biomedicines. 2024 Aug 16;12(8):1874. doi: 10.3390/biomedicines12081874 (PMC11351342; doi:10.3390/biomedicines12081874)
Supplement: Supplementary file 1 [file biomedicines-12-01874-s001.zip › Supplementary Figure/Figure S8/Leaveoneout plot of MR analysis for TL to ubm-b-1628.pdf]

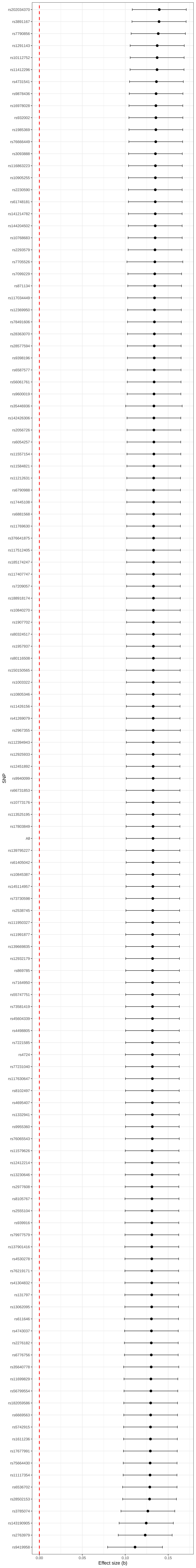
rs202034370
rs3891167
rs7790856
rs1291143
rs10112752
rs11412296
rs4731541
rs9878436
rs16978028
rs932002
rs1985369
rs76666449
rs3093888
rs116863223
rs10905255
rs2230590
rs61748181
rs141214782
rs144204502
rs10768683
rs2293579
rs7705526
rs7099229
rs871134
rs117034449
rs12369950
rs78491606
rs28363070
rs28577594
rs9398196
rs6587577
rs56061761
rs9600019
rs35446936
rs142426306
rs2056726
rs6054257
rs11557154
rs11584821
rs11212631
rs6790988
rs17445108
rs6881568
rs11769630
rs376641875
rs117512405
rs185174247
rs117407747
rs7209057
rs188918174
rs10840270
rs1907702
rs80324517
rs1957937
rs80116508
rs150150565
rs1003322
rs10805346
rs11426156
rs41269079
rs2967355
rs112394943
rs12925933
rs12451892
rs9940099
rs66731853
rs10773176
rs113525195
rs17803849
All
rs139795227
rs61405042
rs10845387
rs145114957
rs73730598
rs2538745
rs111950327
rs11991877
rs139669835
rs12932179
rs869785
rs7164950
rs55747751
rs73581419
rs45604339
rs4498805
rs7221585
rs4724
rs77231040
rs117630647
rs8102497
rs4695407
rs1332941
rs9955360
rs76065543
rs11579626
rs12412214
rs13230646
rs2977608
rs8105767
rs2555104
rs939916
rs79977579
rs137901416
rs4530278
rs76219171
rs41304832
rs131797
rs13062095
rs611646
rs4743037
rs2276182
rs6776756
rs35640778
rs11699829
rs56799554
rs182059586
rs6669563
rs5742915
rs1611236
rs17677991
rs75664430
rs11117354
rs6536702
rs28502153
rs3785074
rs143190905
rs2763979
rs9419958
SNP
0.00
0.05
0.10
0.15
Effect size (b)
